# Laser Additive Manufacturing of Anti-Tetrachiral Endovascular Stents with Negative Poisson’s Ratio and Favorable Cytocompatibility

**DOI:** 10.3390/mi13071135

**Published:** 2022-07-18

**Authors:** Ke Chen, Haoran Wan, Xiang Fang, Hongyu Chen

**Affiliations:** 1 Key Laboratory of Impact and Safety Engineering of Ministry of Education of China, Ningbo University, Ningbo 315211, China; billke1995@gmail.com (K.C.); jacksonwan0706@hotmail.com (H.W.); fangxiang@nbu.edu.cn (X.F.); 2 Department of Vascular Surgery, Second Xiangya Hospital, Central South University, Changsha 410011, China

**Keywords:** laser additive manufacturing, Poisson’s ratio, anti-tetrachiral stents, biocompatibility

## Abstract

Laser additive manufacturing (LAM) of complex-shaped metallic components offers great potential for fabricating customized endovascular stents. In this study, anti-tetrachiral auxetic stents with negative Poisson ratios (NPR) were designed and fabricated via LAM. Poisson’s ratios of models with different diameters of circular node (DCN) were calculated using finite element analysis (FEA). The experimental method was conducted with the LAM-fabricated anti-tetrachiral stents to validate their NPR effect and the simulation results. The results show that, with the increase in DCN from 0.6 to 1.5 mm, the Poisson ratios of anti-tetrachiral stents varied from −1.03 to −1.12, which is in line with the simulation results. The interrelationship between structural parameters of anti-tetrachiral stents, their mechanical properties and biocompatibility was demonstrated. The anti-tetrachiral stents with a DCN of 0.9 mm showed the highest absolute value of negative Poisson’s ratio, combined with good cytocompatibility. The cytocompatibility tests indicate the envisaged cell viability and adhesion of the vascular endothelial cell on the LAM-fabricated anti-tetrachiral auxetic stents. The manufactured stents exhibit great superiority in the application of endovascular stent implantation due to their high flexibility for easy maneuverability during deployment and enough strength for arterial support.

## 1. Introduction

Nowadays, cardiovascular, cerebrovascular, and peripheral vascular diseases have become the causes of death with the highest mortality rates [1]. Atherosclerosis is an important pathophysiological process that causes cardiovascular and cerebrovascular diseases and peripheral vascular diseases [2]. With the generation of atheromatous plaque and accumulation in the arterial intima, the vascular lumen becomes narrow, leading to a reduced blood flow through the blood vessels [2]. This can cause ischemic symptoms in the tissues supplied by the corresponding arteries, with different manifestations according to the branches of the artery and the degree of stenosis, including coronary artery disease, stroke, peripheral arterial disease, and renal artery disease [3]. At present, PTA (percutaneous transluminal angioplasty) has become an important method for treating arteriosclerosis obliterans [4]. The use of a balloon catheter to puncture the diseased artery for treatment is simpler and less risky than traditional medical and surgical treatment, and can partially replace bypass surgery [5]. However, the long-term patency rate of diseased vessels is relatively low due to factors such as elastic recoil, intimal tear, dissection, restenosis, and the progression of arteriosclerosis itself after balloon dilatation alone [6]. Concomitant stent implantation during the procedure can avoid vessel elastic recoil after PTA, residual stenosis, and thromboembolic problems resulting from dissection after application of balloon dilatation [7]. In-stent restenosis is the phenomenon of re-occlusion of the vessel after vascular stent implantation [7,8]. Although vascular stent implantation can support stenotic vessels and prevent elastic recoil of vessels, the vascular wall easily causes inflammatory reaction, intimal hyperplasia, and other problems after stent expansion through rebound, damage, etc. [9], thereby resulting in in-stent restenosis. In-stent restenosis is the main failure form of vascular stents and is a key problem to be solved urgently in vascular stent implantation techniques [9]. Therefore, solving the challenge of in-stent restenosis has become the key scientific issue for vascular stent implant technology [10]. Bare metal stents are prone to causing physical damage to the vessel wall, and non-degradable metal stents may also cause immune rejection in the body [11]. Drug-eluting stents delay in-stent restenosis by adding drugs that inhibit the proliferation of vascular endothelial cells, such as paclitaxel, rapamycin, and their derivatives, on the basis of bare metal stents, but cannot fundamentally solve the problem of restenosis [12,13]. Several studies have shown that the structural design of stents plays an important role in vascular endothelial cell proliferation [14] and long-term stent patency [15,16]. There is also study reporting that stents with negative Poisson’s ratio structures provide new clinical application prospects [17].

The negative Poisson’s ratio structure has a unique tensile/compressive behavior; that is, both transverse strain and longitudinal strain are positive or negative when subjected to uniaxial tension or compression. Structures with negative Poisson’s ratios generally possess high fracture toughness, shear modulus, cracks resistance, and indentation resistance [18]. Modified materials have a surface composition and morphology intended to interact with biological systems and cellular functions. Not only does surface chemistry have an effect on material biological response, surface structures of different morphology can be constructed to guide a desirable biological outcome [10]. In recent years, with intense research on negative Poisson’s ratio structures, many typical negative Poisson’s ratio structures, such as concave hexagonal structure, chiral structure, arrow structure, and rotating quadrangular structure, have been proposed successively [18]. Materials and structures with negative Poisson’s ratios have good application prospects in biomedicine, aerospace, shock absorption and sound insulation, and energy absorption buffering [19]. The application of a negative Poisson’s ratio structure on vascular stents can simultaneously reduce the axial radial size when the vascular stents are compressed before implantation, which is of benefit for minimally invasive implantation. When expansion is performed after implantation, the axial radial size is simultaneously increased, which contributes to the high-precision positioning of the vascular stent. Even though structures of endovascular stents with negative Poisson’s ratios have been designed [20], it is still difficult to manufacture these stents due to their high complexity in configuration and small size. In other words, it is hard to fabricate the small, complexly shaped endovascular stents with negative Poisson’s ratios using traditional manufacturing methods.

In recent years, laser additive manufacturing (LAM) has been developed to manufacture metallic structural parts directly from powder materials [21]. In the work of Roxanne Khalaj et al. [22], coronary stents were manufactured by 3D printing. Weijian Hua et al. [23], showed the 3D printing method to also be a powerful tool for stent manufacturing. Kaitlyn Chua’s work also illustrates how the field of 3D printing and biomedicine can create more innovative devices and products [24]. For 3D printing, the manufacturing structure is already very simple. We can design and manufacture a new honeycomb structure with irregular nodes and pillars [25] and can also use alloy to manufacture a bionic crab claw structure [26]. Laser powder bed fusion (LPBF) is a popular LAM technique capable of precisely preparing complex-shaped parts based on a completely melted mechanism [27]. Its typical “layer-by-layer” fabricating process enables the rapid manufacturing of metallic parts with complex geometries whose preparation is either too tedious or impossible by conventional methods such as casting or forging followed by machining [28]. It has been demonstrated that the LPBF is capable of fabricating negative Poisson’s ratio structures with high precision successfully [29]. LPBF enables the production of highly complex and tailor-performed endovascular stents for patients. Appropriate deformability and biological performance of vascular stents are important factors for the successful implantation of vascular stents and ensuring the restoration of blood flow patency at the vascular stenosis.

In this study, endovascular stents with negative Poisson’s ratios were designed and fabricated via LPBF using 316L stainless steel as the raw material. When the lesion involves a branching vessel, membrane-coated stents could insulate the flow and lead to branch ischemia, whereas a bare metal stent supports the vessels without blocking the flow of branching vessels, though it may cause intimal injury. It depends on the physical situation, and may do more good than harm. Poisson’s ratios of models with different diameters of circular node (DCN) were calculated using finite element analysis (FEA). The stress concentration of a model with different DCN during compressive deformation was analyzed. A quantified method of structural optimization of endovascular stents with negative Poisson’s ratios was proposed. Then, the cytocompatibility tests were conducted on the LPBF-fabricated endovascular stents. The cell viability and adhesion of the umbilical vein endothelial cells on the LPBF-fabricated anti-tetrachiral auxetic stents were investigated. The stent size of this study was mainly based on the diameters of the femoral artery and iliac artery, whose normal values are 7–12 mm. This study shall be a first step toward the structural design and manufacturing of endovascular stents with a negative Poisson’s ratios which fulfill predestined biocompatibility.

## 2. Materials and Methods

### 2.1. Simulation

**Figure 1:**
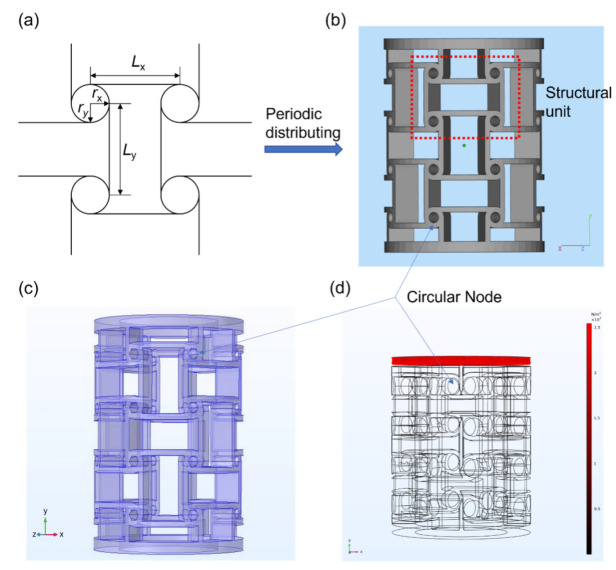
(**a**) Effect of node radius on Poisson’s ratio at the same displacement distance; (**b**) three-dimensional model of anisotropic anti-tetrachiral; (**c**) three-dimensional model in FEM software; (**d**) schematic diagram of boundary conditions.

The geometrical configuration of an anisotropic anti-tetrachiral cell with elliptical nodes is shown in Figure 1a. The geometrical parameters of unit cell *L*_x_, *L*_y_, r_x_, and r_y_ represent the ligament length and radius of the elliptical node along x and y-directions; t represents the wall thickness of the ligaments and nodes; and h is the thickness of the unit cell along the direction perpendicular to the *x–y* plane. Periodically distributing the anisotropic anti-tetrachiral cell into a cylinder so that a model of anti-tetrachiral endovascular stents can be obtained. Finite element analysis using ABAQUS was carried out to predict the mechanical behavior of proposed anti-tetrachiral stent.

The model Figure 1c—whose strut size ratio *L_x_*/*L_y_* was set to 2, and the thickness of the strut was 0.9 mm—was meshed using approximately 200,000 tetrahedral elements. The used mechanical properties of the constituent material of stent for FEA simulation are given in Table 1. Boundary conditions of the unit cell are shown in Figure 1d. When the model was compressed along z-direction, a uniform displacement Uz was imposed on the upper surface of the plate and the displacement of the bottom surface in z-direction was set to zero, and both of the plates could move in the *x*–*y* plane; other surfaces were unconstrained. Variables used to calculate the Poisson ratio are shown in Figure 1b, where Δ*y* is the displacement of the trace surface in the *y* axis and Δ*z* is the displacement in the *z* axis. From the total displacement contour plot, it could be found that all the lateral overhanging struts had the same deforming tendency; moreover, the newly modified re-entrant structure was axisymmetric against the vertical center line, and hence, one end of the strut was chosen to trace the deformation in *y* and *z* axes, and the deformation in the *y*–*z* plane can be used to calculate the Poisson ratio of the structure because of the feasibility of geometric symmetry. The initial lengths of the model in *y* and *z* axes were measured by computer aided design (CAD) software and denoted as *l_y_* and *l_z_*, respectively. Then, the strains in two directions can be calculated using Equation (1) and (2) below.

(1) $$\varepsilon_{\mathrm{tr}ansverse}=\Delta y/l_{y}$$

(2) $$\varepsilon_{\mathrm{ver}tical}=\Delta z/l_{z}$$

Then the Poisson ratio can be obtained using Equation (3) below:

(3) $$\upsilon =\varepsilon_{\mathrm{transverse}}/\varepsilon_{vertical}$$

### 2.2. LPBF Process

**Figure 2:**
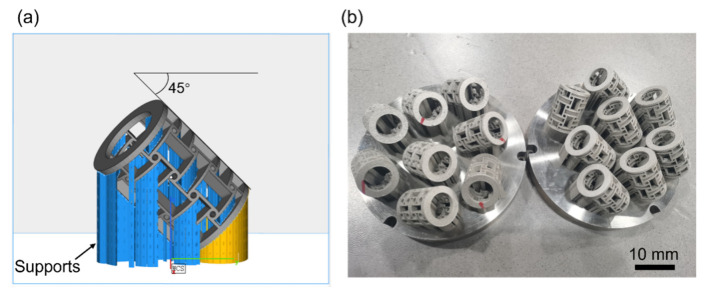
(**a**) Schematic diagram of the support model; (**b**) as-fabricated anti-tetrachiral stents.

The raw material 316L with a spherical shape and a particle range of 15–53 μm was applied in the LPBF process. The 316L powder used was from CarTech. Its UNS Number is S316003. The main specification parameters of 316L: carbon (0.03%), manganese (2.00%), phosphorus (0.045%), sulfur (0.030%), silicon (1.00%), chromium (16.00 to 18.00%), nickel (10.00 to 14.00%). The LPBF processing system consisted of an IPG YLR-500-SM ytterbium fiber laser with a power of ~500 W and a spot size of 70 μm, a SCANLAB hurry scan 30 scanner, an automatic powder spreading apparatus, and a computer control system for LPBF process. The entire process was conducted in an argon atmosphere, and sixteen specimens with dimensions of *ϕ* 9 mm × 12 mm were fabricated layer by layer using laser powder of 160 W, laser scan speed of 800 mm/s, layer thickness of 30 μm, and hatching space of 50 μm. Anti-tetrachiral stents with different radii of node (0.6, 0.9, 1.2, 1.5 mm) were fabricated using the same laser processing parameters. In order to improve the processing quality of the fabricated anti-tetrachiral stents, the model was titled 45 degrees with the substrate and supports were used to ensure the successful fabrication of the stents, as shown in Figure 2a. The as-fabricated anti-tetrachiral stents are shown in Figure 2b.

### 2.3. Experimentation

In order to confirm that the anisotropic anti-tetrachiral cells with elliptical nodes conformed to the simulation results, we prepared samples of each model to be tested experimentally through the instrument. We used four groups of experiments for control comparison: (1) 500 mm/s laser scanning speed and 130 W laser power; (2) 500 mm/s laser scanning speed and 170 W laser power; (3) 1000 mm/s laser scanning speed and 130 W laser power; (4) 1000 mm/s laser scanning speed and 170w laser power. Finally, the best process parameters were selected after four groups of specimens were compared and analyzed: 1000 mm/s laser scanning speed and 170 W laser power. The tests of the Poisson ratio were performed on a Hysitron Tl Premier nanoindenter. By means of matrix punches, the same constraints as in the simulation were applied to the upper surfaces of the specimens in sample navigation, and a pressure of 1000 N was applied to the upper surface, and they were compressed downward by 6 mm at the same time, and the displacement in the y-direction and the displacement in the x-direction of the middle point of the model were calculated by the instrument, and the Poisson ratio of the model could be obtained by dividing the y-direction displacement by the x-direction displacement, and comparing that with the simulation results.

### 2.4. Biocompatibility Test

To investigate the cell viability and adhesion on LPBF-fabricated endovascular stents with negative Poisson’s ratios, polyethylene as a negative control and polyurethane (Sigma-Aldrich, Burlington, MA, USA, 81367, 5 g) as a positive control were obtained according to ISO 10993-12. Human umbilical vein endothelial cells (HUVEC) were cultured in the stents, and the Cell Counting Kit-8 (CCK-8, Dojindo Molecular Technologies, Rockville, MD, USA) assay was used to quantitatively investigate the cytotoxicity of stents [30]. The procedure was as follows: Place the stent in a freeze-dryer at −80 °C; dry for 24 h; then transfer it to a sterile 96-well plate. Use a sterile PBS solution and absolute ethanol to soak the stent for 30 min each. Then, suck away the liquid, place the stent under ultraviolet lamp for irradiation for 24 h for sterilization, and then obtain a sterile printed stent for future use. HUVECs were quantitatively seeded on stents at 1 × 10^5^ cells/mL and supplemented with an appropriate amount of complete medium, and 96-well plates were transferred to a CO_2_ incubator for constant temperature culture, and the medium was changed every day. According to GB/T16886.5-ISO16886-5 Biological evaluation criteria for medical devices [31], polyurethane was selected as a positive control, and polyethylene was selected as a negative control [32]. Pure CCK reagent and endothelial cell medium (ECM) medium were made into a CCK assay reagent at a ratio of 1:10 for future use. We used a tweezer to transfer the cell scaffold construct to be tested inoculated with endothelial cells into a 96-well plate of blank control group previously cultured with the same number of cells; immediately added 110 μL of prepared sterile CCK detection reagent into the experimental group, negative control group, and positive control group at 37 °C; and then transferred the well plate into a CO_2_ incubator for incubation for 3 h. Then, we used a pipette to transfer the liquid in the well plate into another clean well plate, covered the well plate, and transferred it into a microplate reader. We selected the incident wave length as 490 nm, and recorded the measured OD value. Cell survival rate = (OD value of experimental group/OD value of control group) × 100%.

LPBF-fabricated endovascular stents with HUVECs segments were bisected longitudinally to expose the lumen surface and photographed. Both halves of the stents were rinsed in sodium phosphate buffer (pH 7.4) and were then dehydrated in a graded series of ethanol–water. After critical point drying, the tissue samples were mounted and sputter-coated with gold. The samples were visualized using a scanning electron microscopy (SEM, TESCAN, Mira4, Brno, Czech Republic) at 10 keV.

## 3. Results and Discussion

Based on the research on the in-plane deformation behaviors of the hexachiral honeycomb structures demonstrated in literatures, the deformation behavior of anti-tetrachiral structures with elliptical or round nodes under in-plane loading was assumed as ligament-bending deformation mode, which can be analyzed using Euler–Bernoulli beam bending theory [20]. When the node rotates by an angle *φ*, the strains *ε_x_* and *ε_y_* along the *x* and *y*-directions can be expressed as:

(4) $$\varepsilon_{x\;}=\frac{{2(r}_{y}\;-\;\frac{t}{2})\varphi }{L_{x}}$$

and

(5) $$\varepsilon_{y}=\frac{{2(r}_{x}\;-\;\frac{t}{2})\varphi }{L_{y}}$$

Accordingly, the in-plane Poisson ratio *ν_xy_* can be calculated as:

(6) $$\nu_{\mathrm{xy}}=-\frac{\varepsilon_{y}}{\varepsilon_{x}}=-\frac{{2(r}_{x}\;-\;\frac{t}{2}{)L}_{x}}{{2(r}_{y}\;-\;\frac{t}{2}{)L}_{y}}$$

when round nodes are used in the anti-tetrachiral stents, i.e., *r_x_ = r_y_,* the *ν_xy_* can be calculated as $-\frac{L_{x}}{L_{y}}\;$= −γ, which is a constant. Thus, theoretically, the variation of the size of the nodes has no influence on the Poisson ratio of the single in-plane anti-tetrachiral structure. However, after periodically distributing the in-plane anti-tetrachiral structure into three-dimensional anti-tetrachiral stent, its Poisson’s ratio is not a constant and depends on the size of the nodes, as shown in the following.

**Figure 3:**
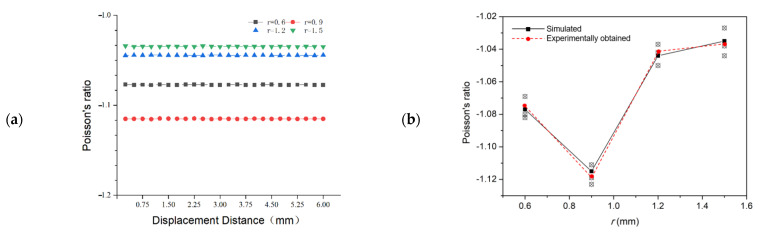
(**a**) Effect of node radius on Poisson’s ratio at the same displacement distance. (**b**) Comparison of experimentally obtained Poisson’s ratio with simulation results.

**Figure 4:**
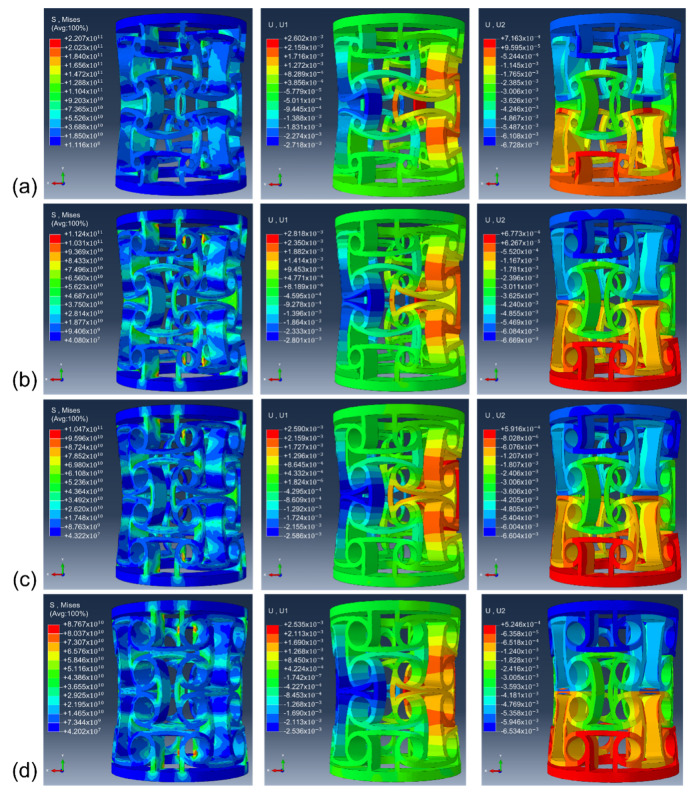
Physical field at each node radius, displacement field in x-direction and displacement field in y-direction: (**a**) r = 0.6 mm;(**b**) r = 0.9 mm; (**c**) r = 1.2 mm; (**d**) r = 1.5 mm.

Figure 3a shows the effect of the node radius on the Poisson ratio of the anti-tetrachiral stent under same displacement distance and the comparison between experimentally obtained and simulated results. It is obvious that all the anti-tetrachiral stents exhibited a negative Poisson ratio; the absolute value of the Poisson ratio increased from 1.077 to the highest value of 1.115 with an increase in node radius from 0.6 to 0.9 mm; then the value decreased to 1.044 as the node radius further increased to 1.2 mm, and finally, to and 1.035 for 1.5 mm. The experimentally obtained Poisson ratio of the anti-tetrachiral stent shows high agreement with the simulated results, as shown in Figure 3b. In order to reveal the influencing mechanism of node radius on the absolute value of Poisson’s ratio of designed anti-tetrachiral stent, the Mises stress field and displacement field of the stent along x and y-directions after compressive deformation were analyzed, as shown in Figure 4. One can see that, after periodically distributing the in-plane anti-tetrachiral structure into the three-dimensional anti-tetrachiral stent, each node of stents is not only subjected to the vertical pressure, but also subjected to the pressure given radially during compression, which causes the inward collapse of the single anti-tetrachiral structure [20]. Table 2 shows the horizontal and vertical displacements of the intermediate nodes of the model for different node radii. The Poisson ratio of the model is calculated by dividing the horizontal displacement by the value of the vertical displacement during the simulation analysis. One can see that the inward collapse displacement (horizontal displacement) of the model with a node radius of 0.9 mm is the largest among the four models, and the evolution of inward collapse displacement agrees with variation in Poisson’s ratio for the four given models. This is to say, the Poisson ratio depends on the inward collapse displacement of models during compressive deformation. When a different node radius is designed for the single anti-tetrachiral structure, the Mises stress and resulting horizontal and vertical displacements are different, so that the responses of different regions and nodes to the pressure and stress can be different (Figure 4). In the case of choosing the same node, the maximum stress in the 0.9 mm radius model was 5.747 × 10^11^, and the stress in the rest of the models decreased with the increase in the node radius. It can be seen in the data that the 0.9 mm model was subjected to the highest stress, and the variations in stress values for the four models are also in accordance with the law of Poisson’s ratio variation. Therefore, the Poisson ratio in different anti-tetrachiral stents with different node radii cannot be a constant. When conducting compression via step-by-step mode, obviously, there is a small fluctuation on Poisson’s ratio for all the anti-tetrachiral stent models. One can speculate that the rings at the top and bottom inhibit the overall shrinkage and have a certain impact on the Poisson ratio. This gives rise to a speculation that the varied negative Poisson’s ratio in anti-tetrachiral stents can be influenced by the suppression effect of the top and bottom rings. The following study shows the simulated results of the model whose original top and bottom rings were removed, so that the suppression effect of rings on the Poisson ratio of anti-tetrachiral stents can be revealed.

**Figure 5:**
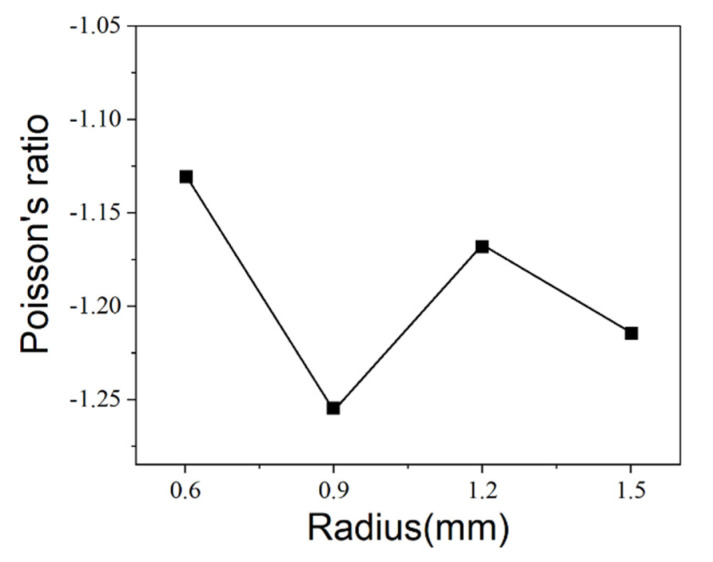
Poisson’s ratio for the four models without rings.

**Figure 6:**
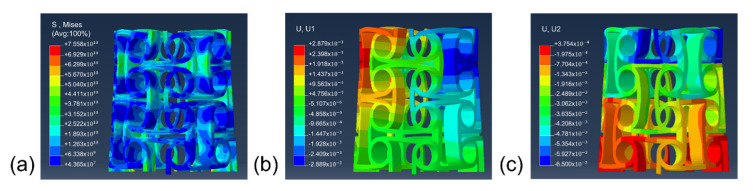
Physical fields of the 1.5 mm model without rings: (**a**) Mises stress field; (**b**) displacement field along the *x*-direction; (**c**) displacement field along the y-direction.

During the study of deformation behavior of anti-tetrachiral structures with round nodes, we proposed the hypotheses: (1) nodes (or circles) are considered rigid; (2) internal forces oriented in a direction perpendicular to the externally applied stress vanish; (3) internal forces are dictated by the observed kinematic behavior; (4) axial and shear deformations of the ligaments are neglected; (5) all deflections are small. This was similar to the work performed by Prall and Lakes [2]. The deformations of the anti-tetrachiral structures with round nodes under uniaxial tensile loading conditions were assumed as ligament bending dominated, and the shearing and tension deformation of the ligaments were not included. We speculate that the upper and lower rings limit the variation of the overall Poisson ratio. Figure 5 shows the analysis of the Poisson ratios of models, which had fixed constraints imposed on their lower bottom surfaces, before and after the ring was removed. The vertical downward displacement of model was set to 6 mm. Based on the simulation results and Table 3, the ring structure inhibits the shrinkage of the whole model. With an increase in the node radius, the inhibition effect became more significant; the absolute values of Poisson’s ratios in the ring-free anti-tetrachiral stents with note radii of 0.6, 0.9, 1.2, and 1.5 mm increased by 3%, 8%, 12%, and 15%, compared to the ring-attached stents. Figure 6 shows the stress and displacement nephogram of the ring-free model with a node radius of 1.5 mm. Combining the above data, it is easy to find that the upper and lower rings have a suppressive effect on the overall deformation of the model. No suppression of the upper and lower rings will make the absolute value of Poisson’s ratio larger for each model, which will make the stresses on the circular nodes more concentrated and more forces in the middle part, leading to more inward collapsing displacements, thereby obtaining larger values of negative Poisson’s ratio.

**Figure 7:**
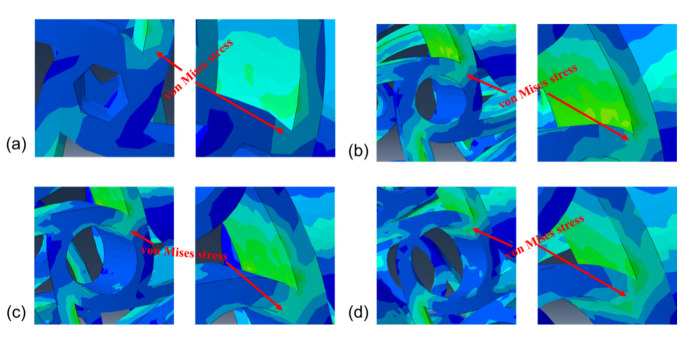
Effect of different radii on stress distribution: (**a**) r = 0.6 mm; (**b**) r = 0.9 mm; (**c**) r = 1.2 mm; (**d**) r = 1.5 mm.

As shown in Figure 3 and Figure 4, the varying of Poisson’s ratio in different anti-tetrachiral stents can be caused by different Mises stress and deformation behavior at the joint fillet position. Thus, the convergence and variation of von Mises stress of anti-tetrachiral models with different node radii were analyzed, as shown in Figure 7. Apparently, the stress variation in anti-tetrachiral stents with a node radius of 0.6 mm during deformation was not that significant compared with that of other stents with larger node radii. As the node radius increases, the stresses acting on each node between each other also increase, and the stress increase was most obvious for the model with r = 0.9 mm. It was also found that the stress concentration most easily occurs at the joint positions between the circular node and other cells. Thus, the circular node in the anti-tetrachiral stent mode plays an important role in the resulting value of negative Poisson’s ratio during the deformation process. The corresponding results show the anti-tetrachiral stent model with an r of 0.9 mm possessed the most uniform stress distribution and largest area of force acting on the joint fillet. This conclusion highly agrees with the results, as shown in Figure 3.

**Figure 8:**
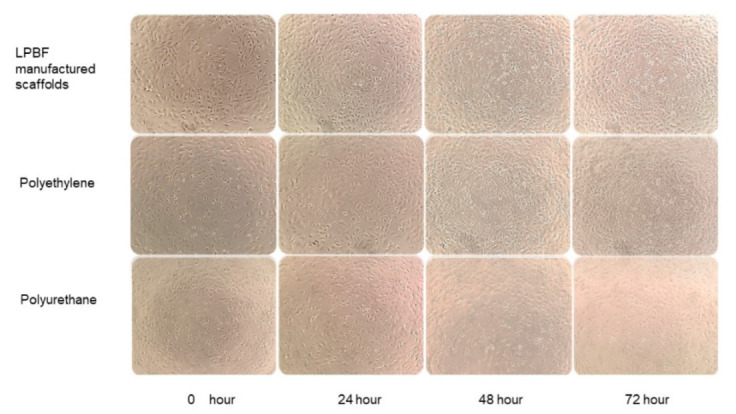
Microscopic view of in vitro cytotoxicity. The morphology of the HUVECs in the stent and polyethylene indicates that the cells were normal. HUVECs in the polyurethane group started to be lysed from 24 h.

Figure 8 shows the human umbilical vein endothelial cells’ (HUVECs) morphology in the LPBF-manufactured scaffolds, and polyethylene was normal and grew well during hours 48 to 72, though there was massive death in the HUVECs co-culture with liquid extracts of polyurethane during hours 48 to 72. According to GB/T 16886.5-2017 qualitative morphological grading of cytotoxicity of extract (Table 4), the LPBF-manufactured scaffolds and polyethylene showed no cell lysis and no reduction in cell proliferation during hours 24 to 72. By comparison, the polyurethane showed obvious lysis of HUVICs. Nearly complete lysis of cell layers took place during hours 24 to 72. According to the toxicity grading method of United States pharmacopeia [33], the relative growth rates (RGR) value evaluation criteria for the cytotoxicity grade of the system, the polyethylene, and LPBF-fabricated scaffolds, the RGR was >95% during the process of cell co-culture, the cytotoxicity was grade 0, and there was no significant difference in the HUVEC counts of the groups (*p* > 0.05). The RGR of polyurethane was 48% at 24 h, and almost zero from 48 h to 72 h, so the cytotoxicity grade could be determined at cytotoxicity grade III, and there was significant difference in the HUVEC counting between polyurethane and LPBF-manufactured scaffolds (*p* < 0.05). The results showed that the cultured cells showed a good growth state on the LPBF-fabricated scaffolds, and the tissue-engineered scaffold constructed after LPBF fabrication of the material had good biocompatibility, a small proportion of apoptosis, and less cytotoxicity, about the same cytotoxicity compared with MIM 316L stents [34].

**Figure 9:**
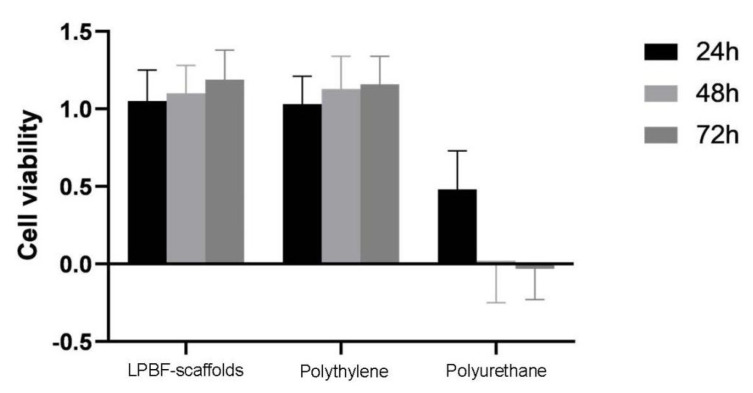
Cytotoxic experiments indicating cellular viability of HUVECs treated with liquid extracts of the LPBF-manufactured scaffolds, polyethylene, and polyurethane at 24, 48, and 72 h. The viabilities of the LPBF-manufactured scaffolds and polyethylene group were higher than 95%, and that of polyurethane was lower than 49%.

**Figure 10:**
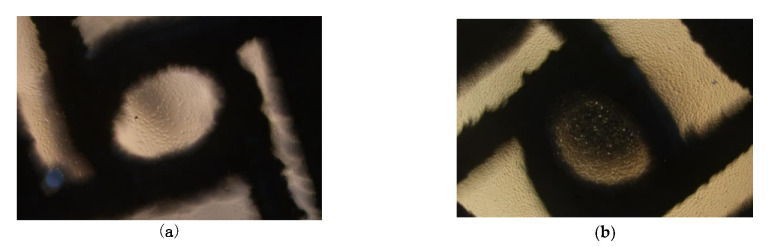
LPBF-manufactured scaffold co-cultured with HUVICs under light microscope: (**a**) co-cultured for 3 days; (**b**) co-cultured for 5 days.

**Figure 11:**
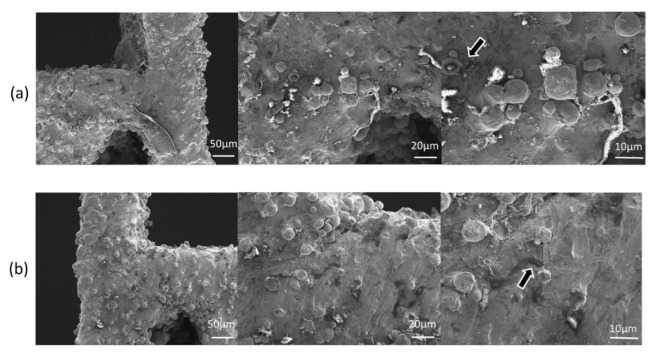
SEM of LPBF-manufactured scaffold loaded with randomly seeded HUVECs. The HUVECs (black arrow) appear to have a spread morphology with cord-like muti-cellular formations. (**a**) Co-cultured for 3 days; (**b**) co-cultured for 5 days.

Figure 9 shows the cell viability and adhesion of the HUVECs on the LPBF-manufactured scaffolds with node radius of 0.9 mm as compared to the control. The cell viability of the LPBF-manufactured scaffold was not significantly different from that of the negative control (polyethylene), and significantly different from the positive control (polyurethane), indicating the excellent biocompatibility of the LPBF-manufactured scaffold. The LPBF-manufactured scaffold co-cultured with HUVICs is shown via light microscopy in Figure 10. The morphologies of the cell adhesion on to the surfaces of the LPBF-manufactured stents after cell culturing for 3d and 5d are shown in Figure 11. The HUVECs adhered to the surfaces of the stents and exhibited a polygonal and full spreading shape; HUVECs are scattered sporadically after culturing in 3d in Figure 11a, indicating that the cells were compatible with the surfaces and grew in a healthy way. By increasing cell culture time to 5d, the cells spread across the surfaces of the stents and covered all of the surface area, as shown in Figure 11b. Overall, the HUVECs on the surfaces of the LPBF-manufactured stents exhibited healthy morphology, attachment, and spreading, as indicated by the flourishing growths of filopodia with widely extended microspikes solidly attached to the surfaces of the scaffolds; see the highly magnified image in Figure 11b. It should be noted that the cell adhesion density in relation to the anti-tetrachiral stents was lower than that of the control. Based on Schulz’s research, HUVECs were reported to favor surfaces with roughness within a few micrometers. The surface roughness of the SLM-manufactured was usually much greater than a few micrometers [36], which would have significantly affected the cells’ seeding on these surfaces, making it more difficult for the cells to settle on the surfaces (Figure 11b). This raises a challenge for the LPBF process: the surface roughness of an LPBF-manufactured product should be effectively reduced, and any partially melted metal particles deposited on the surfaces of the resultant stents should be removed as well as possible. In this way, the cells can be favored to adhere on the surfaces of the LPBF anti-tetrachiral scaffolds and the vascular lumen will become enlarged, leading increased blood flow through the blood vessels to cure vascular occlusive disease, cardiovascular and cerebrovascular diseases, and peripheral vascular diseases. It should be noted that there is still a long way until use in actual patients for the LPBF-fabricated anti-tetrachiral endovascular stents. This study took the first step to prove cytotoxicity, and in the next step, we will undertake animal experiments—for example, implanting stents into the aortas of the dogs; endothelial cell staining; testing the expression levels of CD31, CD34, CD133, and eNOS; and investigating mechanical fatigue reliability. The ultimate aim is developing a more customizable and advanced way to manufacture vascular stents.

## 4. Conclusions

(1) All given anti-tetrachiral stent models possess a negative Poisson’s ratio when subjected to compression force. The stent with r of 0.9 mm has the largest absolute value of negative Poisson’s ratio. The upper and lower rings of the model have a suppressive effect on the overall shrinkage of the model, and the absolute value of the negative Poisson ratio increases when the rings are removed. However, the existence of the ring structure is not the reason for the Poisson ratio not being a constant.
(2) As the node radius increases, the stress concentration phenomenon appears. Stress concentration causes the absolute value of Poisson’s ratio of the model to become smaller. The stress concentration phenomenon appeared least pronounced on the model with a 0.9 mm radius, followed by those with 0.6, 1.2, and 1.5 mm radii. The node radius of 0.9 mm had a larger absolute value of Poisson’s ratio because of the reduced stress concentration at the nodes and other unit connections.
(3) The cultured cells showed good growth state on the LPBF-fabricated scaffolds. They demonstrated favorable structural, physical, and chemical stability and biocompatibility, indicating their promising potential for application in animal and clinical practice.

## Figures and Tables

**Table 1:** Mechanical properties of the constituent material of the stent for FEA simulation.

Material	Young’s Modulus(E)	Poisson Ratio	Yield Stress	Limit Stress	Limit Nominal Strain	Density
316L stainless steel	196,000 MPa	0.3	205 MPa	5151 MPa	60%	$7.89\;\times \;10^{-9}\;\mathrm{ton}/{\mathrm{mm}}^{3}$

**Table 2:** Horizontal and vertical displacements for different node radii.

Node Radius (mm)	Horizontal Displacement (cm)	Vertical Displacement (cm)
0.6	−0.272	0.673
0.9	−0.280	0.670
1.2	−0.259	0.660
1.5	−0.254	0.653

**Table 3:** Comparison of Poisson’s ratios for models with and without rings.

Radius (mm)	Horizontal Deformation (cm)	Vertical Deformation (cm)	Change Rate (%)
0.6	−0.272(with)	0.673(with)	3
	−0.283(without)	0.719(without)	
0.9	−0.280(with)	0.670(with)	8
	−0.301(without)	0.717(without)	
1.2	−0.259(with)	0.660(with)	12
	−0.297(without)	0.679(without)	
1.5	−0.254(with)	0.653(with)	15
	−0.289(without)	0.650(without)	

**Table 4:** Qualitative morphological grading of cytotoxicity of extract [35].

Level	Extent of Reaction	Morphology of Cultured Cells
0	None	Discrete intracytoplasmic volume, no cell lysis, no reduction of cell proliferation
1	Minor	Not more than 20% of the cells are round, loosely attached, and without cytoplasmic or morphological changes; occasional lysed cells are present; only slight inhibition of cell growth is observed
2	Mild	Not more than 50% of the cells are round, devoid of intracytoplasmic granules, no extensive cell lysis, not more than 50% growth inhibition observable
3	Moderate	Not more than 70% of the cell layers contain rounded cells or are lysed; cell layers not completely destroyed, but more than 50% cell growth inhibition may be observed
4	Severe	Nearly complete or complete destruction of cell layers

## Data Availability

All reasonable requests for materials and data will be fulfilled by the corresponding author of this publication.

## References

[1] Roth G.A., Dwyer-Lindgren L., Bertozzi-Villa A., Stubbs R.W., Morozoff C., Naghavi M., Mokdad A.H., Murray C.J.L. (2017). Trends and Patterns of Geographic Variation in Cardiovascular Mortality Among US Counties, 1980–2014. JAMA.

[2] Lee J.M., Choi K.H., Koo B.-K., Park J., Kim J., Hwang D., Rhee T.-M., Kim H.Y., Jung H.W., Kim K.-J. (2019). Prognostic Implications of Plaque Characteristics and Stenosis Severity in Patients With Coronary Artery Disease. J. Am. Coll. Cardiol..

[3] Libby P., Buring J.E., Badimon L., Hansson G.K., Deanfield J., Bittencourt M.S., Tokgözoğlu L., Lewis E.F. (2019). Atherosclerosis. Nat. Rev. Dis. Primers.

[4] Schneider P.A., Laird J.R., Doros G., Gao Q., Ansel G., Brodmann M., Micari A., Shishehbor M.H., Tepe G., Zeller T. (2019). Mortality Not Correlated With Paclitaxel Exposure: An Independent Patient-Level Meta-Analysis of a Drug-Coated Balloon. J. Am. Coll. Cardiol..

[5] Lookstein R.A., Haruguchi H., Ouriel K., Weinberg I., Lei L., Cihlar S., Holden A. (2020). Drug-Coated Balloons for Dysfunctional Dialysis Arteriovenous Fistulas. N. Engl. J. Med..

[6] Tepe G., Laird J., Schneider P., Brodmann M., Krishnan P., Micari A., Metzger C., Scheinert D., Zeller T., Cohen D.J. (2015). Drug-coated balloon versus standard percutaneous transluminal angioplasty for the treatment of superficial femoral and popliteal peripheral artery disease: 12-month results from the IN.PACT SFA randomized trial. Circulation.

[7] Bausback Y., Wittig T., Schmidt A., Zeller T., Bosiers M., Peeters P., Brucks S., Lottes A.E., Scheinert D., Steiner S. (2019). Drug-Eluting Stent Versus Drug-Coated Balloon Revascularization in Patients With Femoropopliteal Arterial Disease. J. Am. Coll. Cardiol..

[8] Yang X., Yang Y., Guo J., Meng Y., Li M., Yang P., Liu X., Aung L.H.H., Yu T., Li Y. (2021). Targeting the epigenome in in-stent restenosis: From mechanisms to therapy. Mol. Ther. Nucleic Acids.

[9] Byrne R.A., Joner M., Kastrati A. (2015). Stent thrombosis and restenosis: What have we learned and where are we going? The Andreas Grüntzig Lecture ESC 2014. Eur. Heart J..

[10] Slepicka P., Kasalkova N.S., Siegel J., Kolska Z., Bacakova L., Svorcik V. (2015). Nano-structured and functionalized surfaces for cytocompatibility improvement and bactericidal action. Biotechnol. Adv..

[11] Wu X., Yin T., Tian J., Tang C., Huang J., Zhao Y., Zhang X., Deng X., Fan Y., Yu D. (2015). Distinctive effects of CD34- and CD133-specific antibody-coated stents on re-endothelialization and in-stent restenosis at the early phase of vascular injury. Regen. Biomater..

[12] Yin R.-X., Yang D.-Z., Wu J.-Z. (2014). Nanoparticle drug- and gene-eluting stents for the prevention and treatment of coronary restenosis. Theranostics.

[13] Torrado J., Buckley L., Durán A., Trujillo P., Toldo S., Valle Raleigh J., Abbate A., Biondi-Zoccai G., Guzmán L.A. (2018). Restenosis, Stent Thrombosis, and Bleeding Complications: Navigating Between Scylla and Charybdis. J. Am. Coll. Cardiol..

[14] Holy E.W., Jakob P., Eickner T., Camici G.G., Beer J.H., Akhmedov A., Sternberg K., Schmitz K.-P., Lüscher T.F., Tanner F.C. (2014). PI3K/p110α inhibition selectively interferes with arterial thrombosis and neointima formation, but not re-endothelialization: Potential implications for drug-eluting stent design. Eur. Heart J..

[15] Pant S., Limbert G., Curzen N.P., Bressloff N.W. (2011). Multiobjective design optimisation of coronary stents. Biomaterials.

[16] MacTaggart J., Poulson W., Seas A., Deegan P., Lomneth C., Desyatova A., Maleckis K., Kamenskiy A. (2019). Stent Design Affects Femoropopliteal Artery Deformation. Ann. Surg..

[17] Stoeckel D., Bonsignore C., Duda S. (2002). A survey of stent designs. Minim. Invasive Ther. Allied Technol..

[18] Stergaard M.B., Hansen S.R., Januchta K., To T., Smedskjaer M.M. (2019). Revisiting the Dependence of Poisson’s Ratio on Liquid Fragility and Atomic Packing Density in Oxide Glasses. Materials.

[19] Scarpa F., Smith C.W., Ruzzene M., Wadee M.K. (2008). Mechanical properties of auxetic tubular truss-like structures. Phys. Status Solidi (B).

[20] Wu W., Song X., Liang J., Xia R., Qian G., Fang D. (2018). Mechanical properties of anti-tetrachiral auxetic stents. Compos. Struct..

[21] Zhang W., Zhang B., Xiao H., Yang H., Wang Y., Zhu H. (2021). A Layer-Dependent Analytical Model for Printability Assessment of Additive Manufacturing Copper/Steel Multi-Material Components by Directed Energy Deposition. Micromachines.

[22] Khalaj R., Tabriz A.G., Okereke M.I., Douroumis D. (2021). 3D printing advances in the development of stents. Int. J. Pharm..

[23] Hua W., Mitchell K., Raymond L., Godina B., Zhao D., Zhou W., Jin Y. (2021). Fluid Bath-Assisted 3D Printing for Biomedical Applications: From Pre-to Postprinting Stages. ACS Biomater. Sci. Eng..

[24] Chua K., Khan I., Malhotra R., Zhu D. (2021). Additive manufacturing and 3D printing of metallic biomaterials. Eng. Regen..

[25] Yang J., Gu D., Lin K., Wu L., Zhang H., Guo M., Yuan L. (2021). Laser additive manufacturing of cellular structure with enhanced compressive performance inspired by Al–Si crystalline microstructure. CIRP J. Manuf. Sci. Technol..

[26] Ma C., Gu D., Lin K., Dai D., Xia M., Yang J., Wang H. (2019). Selective laser melting additive manufacturing of cancer pagurus’s claw inspired bionic structures with high strength and toughness. Appl. Surf. Sci..

[27] Chen H., Gu D., Xiong J., Xia M. (2017). Improving additive manufacturing processability of hard-to-process overhanging structure by selective laser melting. J. Mater. Process. Technol..

[28] Chen H., Gu D., Deng L., Lu T., Kühn U., Kosiba K. (2021). Laser additive manufactured high-performance Fe-based composites with unique strengthening structure. J. Mater. Sci. Technol..

[29] Xiong J., Gu D., Chen H., Dai D., Shi Q. (2017). Structural optimization of re-entrant negative Poisson’s ratio structure fabricated by selective laser melting. Mater. Des..

[30] Plaza A., Merino B., Del Olmo N., Ruiz-Gayo M. (2019). The cholecystokinin receptor agonist, CCK-8, induces adiponectin production in rat white adipose tissue. Br. J. Pharmacol..

[31] Li C.R., Li L., Cao H.J., Qin L., Long J., Lai Y.X., Wang X.L., Li Y. (2015). In vitro biosafety assessment of PLGA/TCP/Mg porous scaffold for bone regeneration. Session Biomater. Implants..

[32] Bäcker H.C., Wu C.H., Krüger D., Gwinner C., Perka C., Hardt S. (2020). Metal on Metal Bearing in Total Hip Arthroplasty and Its Impact on Synovial Cell Count. J. Clin. Med..

[33] United States Pharmacopeial Convention (1990). Committee of Revision. USP XXII, or, NF XVII, or The United States Pharmacopeia, or, The National Formulary.

[34] Shu C., He H., Fan B.-W., Li J.-H., Wang T., Li D.-Y., Li Y.-M., He H. (2022). Canine implant biocompatibility of vascular stents manufactured using metal injection molding. Trans. Nonferrous Met. Soc. China.

[35] Ablikim Z., Ablikim G., Zhu Q.F., Chen T., Gang W.U., Hua Y.Y., Wang S.H., Hospital C., Pharmacy S.O. (2019). Preparation of five kinds of acellular fish skin matrices and in vitro toxicity analysis. Acad. J. Second Mil. Med. Univ..

[36] Fu X., Liu P., Zhao D., Yuan B., Zhang X. (2020). Effects of Nanotopography Regulation and Silicon Doping on Angiogenic and Osteogenic Activities of Hydroxyapatite Coating on Titanium Implant. Int. J. Nanomed..

